# Electrospun Three-Dimensional Nanofibrous Structure via Probe Arrays Inducing

**DOI:** 10.3390/mi9090427

**Published:** 2018-08-24

**Authors:** Yifang Liu, Ruimin Liu, Xiang Wang, Jiaxin Jiang, Wenwang Li, Juan Liu, Shumin Guo, Gaofeng Zheng

**Affiliations:** 1 Department of Instrumental and Electrical Engineering, Xiamen University, Xiamen 361102, China; yfliu@xmu.edu.cn (Y.L.); wudideyueqiu@stu.xmu.edu.cn (R.L.); jiangjx@xmu.edu.cn (J.J.); cecyliu@xmu.edu.cn (J.L.); 2 School of Mechanical and Automotive Engineering, Xiamen University of Technology, Xiamen 361024, China; wx@xmut.edu.cn (X.W.); xmlww@xmut.edu.cn (W.L.); 3 School of Mathematical Sciences, Xiamen University, Xiamen 361005, China; shumin_guo@xmu.edu.cn; 4 Fujian Provincial Key Laboratory of Mathematical Modeling and High Performance Scientific Computing, Xiamen University, Xiamen 361005, China

**Keywords:** electrospinning, nanofiber, three-dimensional micro structures, probe arrays, induced electrical field

## Abstract

The fast and precise direct-printing of micro three-dimensional (3D) structures is the important development trend for micro/nano fabrication technique. A novel method with probe arrays was built up to realize the controllable deposition of 3D electrospun nanofibrous structures. Firstly, several 3D nanofibrous structures were built on a single probe and 2-, 3-probes, which indicated that the probe height and probe interval played a key role on the 3D structure morphology. Then, different stereo nanofibrous structures based on multiprobe arrays were achieved accurately and the effects of processing parameters, including the probe height, probe interval, applied voltage and flow rate on the deposition behaviors of electrospun nanofiber over the probe arrays were investigated. The deposition area of 3D electrospun nanofibrous structures decreased with the increase of probe interval, applied voltage, and flow rate. Several 3D nanofibrous structures of special shapes including convex, triangle wave, inverted cone and complex curved surface were demonstrated by controlling the configuration of probe arrays and electrospinning parameters. This work provides an effective and simple way for the construction of 3D electrospun nanofibrous structures, which has great potentials in various medical and industrial applications.

## 1. Introduction 

With special advantages, nanofibrous structures have been applied to various industrial fields, such as bio-scaffold, tissue engineering, and composite materials, etc. [1,2,3]. Methods to construct nanofibrous structures include etching, prototyping, polymer/fiber deposition, template synthesis, nanosecond laser processing, self-assembly, and electrospinning [4,5,6,7]. Among these methods, electrospinning, in particular, has been intriguing due to its characteristics of simple process, relatively low cost, high efficiency, and good materials compatibility [8,9,10]. What is more, the electrospun structures have remarkable features compared to those prepared by other technologies, including high surface area-to-volume ratio, small pore size and good biocompatibility, making them more ideal for applications of different industrial fields [11,12,13]. 

Fabrication of two-dimensional (2D) nanofibrous structures using electrospinning has been at its mature age, while there is still a lot to explore for its three-dimensional (3D) counterpart [14,15]. With the increasing demand for different applications, various electrospinning approaches have been investigated and developed to prepare 3D structures according to their practical use [16,17]. A straightforward method is to continuously deposit nonwoven mats with the instable motion of liquid jet. The residual charge on the nanofibrous membrane hinders the deposition of following nanofiber that carries free charge from the nozzle, which blocks the fast fabrication of 3D microstructures [18,19]. Assistant external force should be utilized to promote layer-by-layer deposition of nanofibers to build up 3D structures. The assistant force is also required urgently for the fast fabrication of composite structure from different materials, which becomes the important trend for applications of nanofibers. The as-prepared membranes could be post-processed by tailoring or rolling up to form different patterns [20]. Self-assembled 3D structures could also be achieved by electrospinning process. Loo et al. [21] investigated the nanofibrous 3D hydrogels that were self-assembled from peptide bioinks–lysine-containing hexapeptides. However, most above-mentioned approaches can only fabricate structures with uniform thickness or of certain shape. 

In our previous works, we have fabricated successfully multiloop nanofibrous coils on the plane collector by using the spiral motion of electrohydrodynamic direct-writing charged jet [22], but the 3D structure was deposited randomly. This method cannot be used to build up complex architectures. Tong et al. [23] proposed a novel technology with the alternate use of positive voltage and negative voltage, which attained much better performance than the conventional electrospinning in the view of fiber thickness. However, there is still some limitation on the shape of structures. Then, the template-assisted collection was also introduced to realize layer-by-layer deposition of electrospinning nanofiber, and attracts attention from many researchers, by which the shape and size of 3D microstructures can be adjusted. For instance, Zhang et al. [24] reported the fabrication of 3D fibrous tubes with 3D collecting templates. Mi et al. [25] reported a novel electrospinning setup with a specially designed collector including a separate fiber removal device through which spun fibers were removed without destruction. The collected fibers were of high alignment and desirable thickness. Zhu et al. [26] introduced a “dumbbell” collector into the electrospinning process, and a special 3D macrostructure made of multilayer fibrous membranes was collected. 

In this work, an inducing technique stemming from probe arrays is built up to realize the fabrication of 3D nanofibrous structures; the effects of processing parameters were investigated, 3D structures of different shapes were fabricated.

## 2. Materials and Methods 

**Figure 1:**
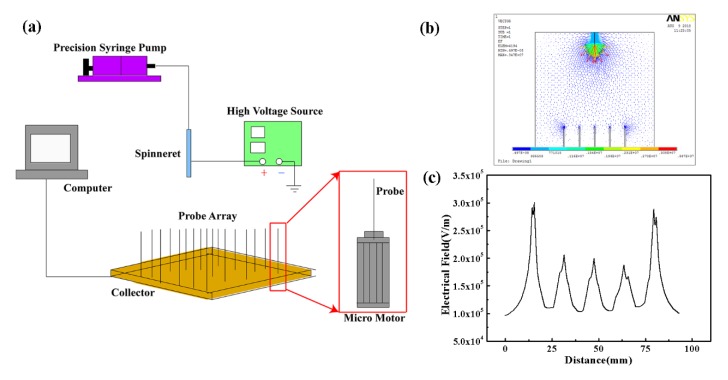
Schematic diagram of the proposed electrospinning setup and simulation of induced electrical field. (**a**) Experimental schematic diagram; (**b**) Electrical field distribution; (**c**) variation curve of electrical field along the line of probe tips (red line in Figure 1b).

An electrospinning setup with induced probe arrays was built up as shown in Figure 1, including a spinneret, a precision syringe pump (Pump 11 Pico Plus Elite, Harvard Apparatus, MA, USA), a high voltage source (DW-SA403-1ACE5, 0~50 kV DC, Dongwen high-voltage power source Ltd., Tianjin, China), a designed collector with probe arrays and a host computer. The high voltage source was utilized to generate a high electric field between the spinneret and the collector. The prepared solution was supplied to the spinneret at a constant flow rate by the precision syringe pump. An aluminum wafer was used as the collector, on which iron probes were placed in arrays to induce the construction of 3D stereo nanofibrous structure. The height of each probe could be adjusted by a micro motor, which was fixed under the collector and controlled by a host computer, thus the electric field could be changed according to the molding demand of 3D nanofibrous structures. The morphology of the 3D nanofibrous structures was recorded by an optical microscope (Mitutoyo, Kanagawa, Japan). The experiments were conducted under the temperature of 20~25 °C and the surrounding humidity of 40%~50% RH.

Poly (Ethylene Oxide) (PEO, M_w_ = 300,000 g/mol, Dadi Fine Chemical Co, Ltd., Changchun, China) solution was used as the electrospinning material in this experiments. To prepare the PEO solution, the powder was dissolved and stirred in a mixture solvent of deionized water and ethanol with volume ratio of 1:1. The ethanol was added to accelerate the evaporation rate and decrease the dielectric constant of solvent, which was contributed to increasing the diameter of nanofibers and promoting the construction of 3D electrospun structures [27,28]. Then, the mixture solution was kept at room temperature for 5 h to ensure full dissolution of polymer powder.

## 3. Results and Discussion

The induced electrical field was simulated using the finite element method, as shown in Figure 1b, and the variation curve of electrical field along the line of probe tips was depicted in Figure 1c. From the simulation results, it could be seen that there was a concentrated electrical field on each tip of the probe array, contributing to the guidance of nanofibers deposited into a designed 3D controllable structure. In this simulation model, the applied voltage, the probe height, and the probe interval were set to be 20 kV, 20 mm, and 10 mm, respectively.

**Figure 2:**
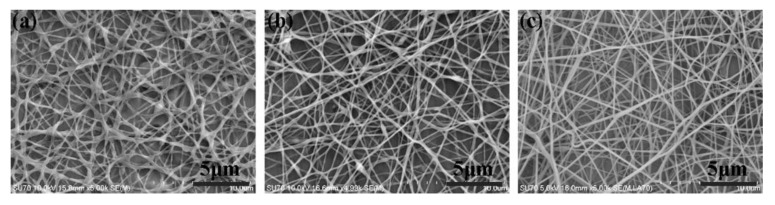
Scanning electron microscope (SEM) images of deposited nanofibers with different solution concentrations. (**a**) 8 wt %; (**b**) 10 wt %; (**c**) 12 wt %. The applied voltage, the flow rate, and the distance between the spinneret and the collector were 20 kV, 300 μL/h, and 15 cm, respectively.

The solution concentration was an important factor that affected the morphology of deposited nanofibers [29], so it was investigated first. As shown in Figure 2, when the solution concentration was below 10 wt %, the solvent could not evaporate completely, thus the nanofibers were easy to gather together or spread out, consisting of some bead structures as well. While the solution concentration increased to 12 wt %, it could be seen from Figure 2c that the deposited nanofibers were much smoother and more uniform, which would contribute to the construction of 3D structures with high mechanical strength and structural stability. However, if the solution concentration was too high, there would be a high viscous tension among deposited nanofibers, resulting in lots of fibers deposited on the probe tips, influencing the uniformity of nanofiber deposition. So, in our following experiments to build various 3D nanofibrous structures on probe arrays, PEO solution with concentration of 12 wt % was utilized.

A series of experiments were carried out to investigate the effects of processing parameters on the deposition of 3D nanofibrous structure. The distance between the spinneret and the collector was fixed to be 15 cm and the deposition time was set to be 15 min. Under the high electrical field between the spinneret and the collector, a charged jet ejected from the Taylor cone and travelled to collector and deposited over the tips of probe array to achieve the 3D microstructures.

**Figure 3:**
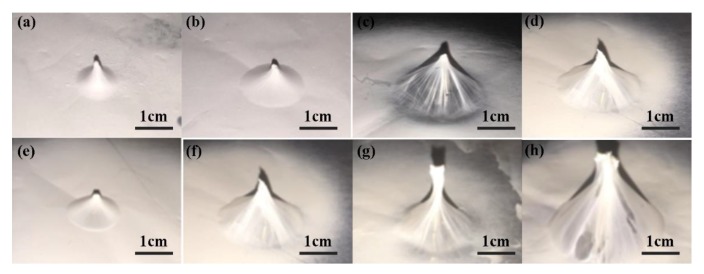
3D nanofibrous structures printed on a single probe. (**a**) 12 kV and (**b**) 20 kV with 5 mm probe height; (**c**) 12 kV and (**d**) 20 kV with 10 mm probe height; (**e**) 5 mm, (**f**) 10 mm; (**g**) 15 mm; (**h**) 20 mm with applied voltage of 20 kV. The flow rate was 300 μL/h.

The 3D nanofibrous structures that printed on a single probe under different voltages were presented in Figure 3a–d. It was obvious that with higher applied voltage, the productivity of nanofibers was higher, thus the fibers were stretched more completely and the thickness of the nanofibrous membrane was more uniform. With the probe height of 5 mm, the thicknesses of fabricated nanofibrous membrane were 156.1 nm and 102.5 nm, respectively, for applied voltage of 12 kV and 20 kV, while they were 51.2 nm and 75.7 nm, respectively, for the probe height of 10 mm. 

Figure 3e–h showed the 3D nanofibrous structures printed on a single probe with probe heights of 5 mm, 10 mm, 15 mm, and 20 mm, respectively. When the probe height was lower than 10 mm, the nanofibers deposited over the tip of probe and formed a conical shape. While it was higher than 10 mm, the nanofibers deposited mainly on the tip of probe. Even for the probe with height of 20 mm, non-uniformity of thickness appeared on the 3D structure, which was not desirable for industrial applications. 

**Figure 4:**
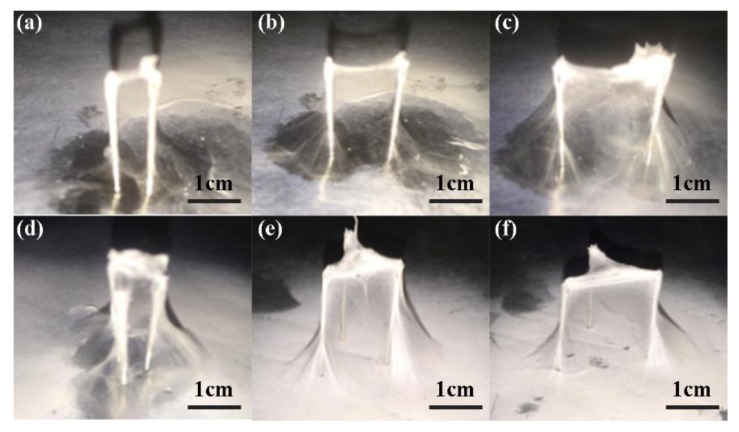
3D nanofibrous structure printed on probe arrays with different intervals; (**a**) 5 mm, (**b**) 10 mm, and (**c**) 15 mm of 2 probes; (**d**) 5 mm, (**e**) 10 mm, and (**f**) 15 mm of 3 probes. The probe height, the flow rate, and the applied voltage were 10 mm, 300 μL/h, and 20 kV, respectively.

Figure 4 showed the structures deposited on 2-, 3-probe arrays with different probe intervals, of which Figure 4a–c presented the probe intervals of 5 mm, 10 mm, 15 mm, respectively, when there were 2 probes, while Figure 4d–f illustrated the structures built on 3 probes. When the probe interval was 5 mm, the deposited nanofibers gathered together without a smooth morphology due to the inducing effects of the probe tips on the electrical field, making it not suitable for the application of 3D structures. With the interval increased, the surface morphology became smoother and more uniform, and stable structures could be obtained among the probes. However, when the probe intervals were too large, each probe would take effect separately, and the structures among the probes were very fragile with a low mechanical strength, or even there might not be any fibers connecting each two probes. From the results, it could be concluded that a proper probe height and probe interval was important for the construction of 3D nanofibrous membrane.

**Figure 5:**
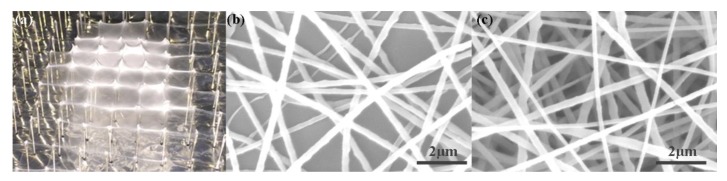
3D nanofibrous structure printed on multiprobe arrays. (**a**) Photograph of 3D nanofibrous structure; (**b**) SEM image of nanofibers deposited among the probes; (**c**) SEM image of nanofibers deposited on the tip of probes. The probe height, probe interval, applied voltage, and flow rate were 20 mm, 10 mm, 25 kV, and 300 μL/h, respectively.

Then, the deposition characteristics of 3D electrospun nanofibrous structures induced by multiprobe arrays were investigated further. Figure 5a showed a 3D structure printed on the probe arrays with height of 20 mm and interval of 10 mm. With uniform probe heights of all the probes, a flat 3D nanofibrous structure could be obtained, of which the majority of nanofibers were gathered on the tip of probes due to the induced electrical field. Scanning electron microscope (SEM) images of nanofibers were presented in Figure 5b,c. It could also be seen that the fiber density on the tip of probes was much higher than that among the probes, which contributed to the construction of nanofibrous structure and enhanced the mechanical strength to maintain a stable 3D shape.

**Figure 6:**
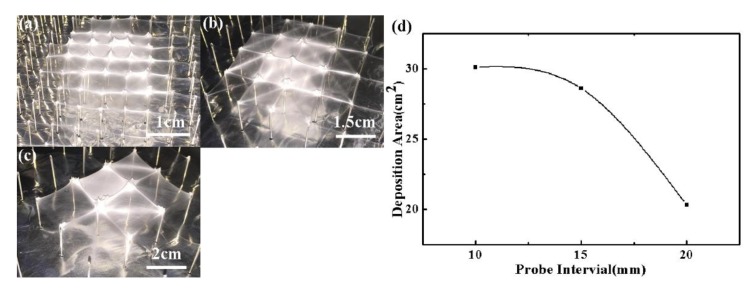
Effect of probe intervals on the printed 3D nanofibrous structures. (**a**) 10 mm; (**b**) 15 mm; (**c**) 20 mm; (**d**) relationship between deposition area and probe interval. The probe height, applied voltage, and flow rate were 20 mm, 25 kV, and 300 μL/h, respectively.

The effects of processing parameters of electrospinning on the deposition of 3D nanofibrous structures were discussed further. Firstly, the effects of probe intervals were investigated, as shown in Figure 6. When the probe interval was 20 mm, the nanofibers were mostly deposited onto the tip of probes just underneath the spinneret and the deposition area was 20.3 cm^2^, far smaller than that with probe interval of 10 mm and 15 mm, because the induced electrical field was not high enough when there was a larger probe interval, which hampered the larger-area deposition of nanofibrous structures. 

**Figure 7:**
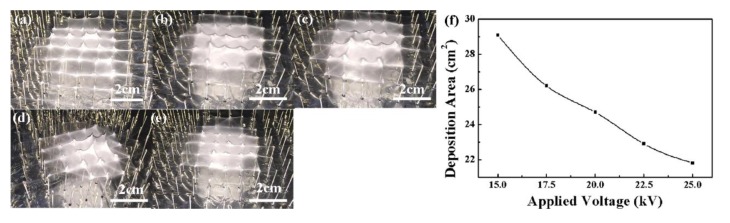
Effect of applied voltages on the printed 3D nanofibrous structures. (**a**) 15 kV; (**b**) 17.5 kV; (**c**) 20 kV; (**d**) 22.5 kV; (**e**) 25 kV; (**f**) relationship between deposition area and applied voltage. The probe height, probe interval, and flow rate were 20 mm, 10 mm, and 300 μL/h, respectively.

**Figure 8:**
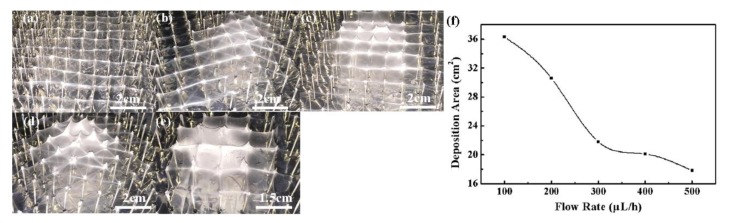
Effect of flow rates on the printed 3D nanofibrous structures. (**a**) 100 μL/h; (**b**) 200 μL/h; (**c**) 300 μL/h; (**d**) 400 μL/h; (**e**) 500 μL/h; (**f**) relationship between deposition area and flow rate. The probe height, probe interval, and applied voltage were 20 mm, 10 mm, and 25 kV, respectively.

Figure 7 showed the relationship between the deposition area and the applied voltage. With applied voltages of 15 kV, 17.5 kV, 20 kV, 22.5 kV, and 25 kV, the deposition area were 29.1 cm^2^, 26.2 cm^2^, 24.7 cm^2^, 22.9 cm^2^, and 21.8 cm^2^, respectively. Figure 8 illustrated the relationship between the deposition area and the solution flow rate with a probe height of 20 mm, a probe interval of 10 mm and an applied voltage of 25 kV. When the flow rate increased from 100 μL/h to 500 μL/h, the deposition area decreased from 36.3 cm^2^ to 17.8 cm^2^. A higher applied voltage and a larger flow rate resulted in a higher initial ejection speed of the charge jet, thus the time for jet in flight was shortened and there was not enough time for the jet to whip in a large area, leading to a smaller deposition area of 3D nanofibrous structures. 

**Figure 9:**
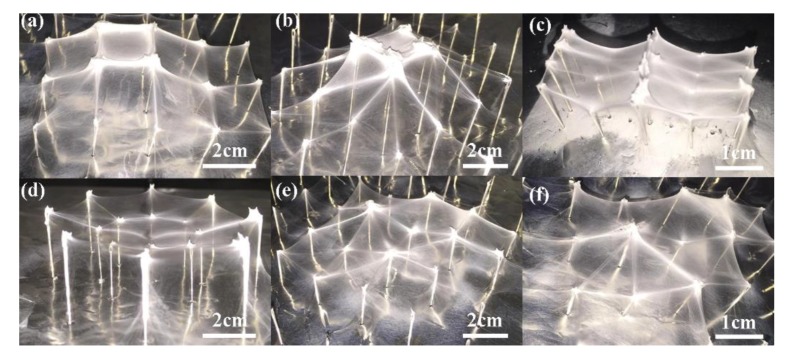
3D nanofibrous structures printed on probe arrays with non-uniform probe heights. (**a**,**b**) Convex; (**c**) triangle wave; (**d**) inverted cone; (**e**,**f**) complex curved surface.

Particularly, more 3D electrospun nanofibrous structures with different special shapes could be obtained by adjusting the probe heights and intervals in a designed configuration. For instance, a typical convex shape could be obtained by raising the probes in the central area of the probe arrays. Similarly, by raising the probes in one row and lowering that in the next row, a triangle wave shape could be formed. By arranging the multiple probes in special arrays to manipulation the distribution of induced electrical field, several 3D structures, such as convex, triangle wave, inverted cone and even complex curved surface with no specific shapes were achieved successfully, as shown in Figure 9. In this way, 3D stereo structures with complex shapes of large area could be obtained in a simple way without designing a fixed-shape template to meet the application requirements of electrospun nanofibers.

## 4. Conclusions

Stereo nanofibrous structures could be obtained by manipulation of the electric field in the electrospinning process. In this work, induced probe arrays were introduced to build the 3D nanofibrous structures with complex shapes. The probe tips provided an inducing electrical field to guide the deposition of electrospun nanofibers, which also enhanced the mechanical strength and stereo stability of constructed 3D structures. The effects of the processing parameters, including the probe height, the probe interval, the applied voltage, and the solution flow rate on the fabrication of 3D nanofibrous structures on multiple probe arrays were investigated. It was found that a moderate probe height and probe interval played an important role for the construction of 3D stereo structures. The deposition areas of 3D nanofibrous structures decreased with the increase of probe intervals, applied voltage, and solution flow rate. By adjusting the probe heights, various 3D nanofibrous structures with special shapes, such as convex, triangle wave, inverted cone, and complex curved surface with areas larger than 20 cm^2^ were obtained. The introduction of induced probe arrays provides an effective method in the controllable deposition of 3D electrospun nanofibrous structures, which will promote its applications in many fields.
